# Should Regional Species Loss Be Faster or Slower Than Local Loss? It Depends on Density‐Dependent Rate of Death

**DOI:** 10.1002/ece3.71162

**Published:** 2025-03-27

**Authors:** Petr Keil, Adam T. Clark, Vojtěch Barták, François Leroy

**Affiliations:** ^1^ Faculty of Environmental Sciences Czech University of Life Sciences Prague Praha ‐ Suchdol Czech Republic; ^2^ Department of Biology University of Graz Graz Austria; ^3^ Department of Evolution, Ecology and Organismal Biology The Ohio State University Columbus Ohio USA

**Keywords:** Allee, extirpation, Janzen–Connell, scaling, sixth mass extinction, species richness

## Abstract

Assessment of the rate of species loss, which we also label extinction, is an urgent task. However, the rate depends on spatial grain (average area *A*) over which it is assessed—local species loss can be, on average, faster or slower than regional or global loss. Ecological mechanisms behind this discrepancy are unclear. We propose that the relationship between extinction rate and *A* is driven by a classical ecological phenomenon: density‐dependent mortality. Specifically, we hypothesize that (i) when per‐individual probability of death (*P*_death_) decreases with the number of individuals in a region *N* (i.e., negative density dependence), per‐species extinction rate (*Px*) should be high at regional grains and low locally. (ii) In contrast, when *P*_death_ increases with *N* (i.e., positive density dependence), *Px* should be low regionally but high locally. (iii) Total counts of extinct species (*Ex*) should follow a more complex relationship with *A*, as they also depend on drivers of the species‐area relationship (SAR) prior to extinctions, such as intraspecific aggregation, species pools, and species‐abundance distributions. We tested these hypotheses using simulation experiments, the first based on point patterns and the second on a system of generalized Lotka–Volterra equations. In both experiments, we used a single continuous parameter that moved between the negative, zero, and positive relationship between *P*_death_ and *N*. We found support for our hypotheses, but only when regional species‐abundance distributions were uneven enough to provide sufficiently rare or common species for density dependence to act on. In all, we have theoretically demonstrated a mechanism behind different rates of biodiversity change at different spatial grains, which has been observed in empirical data.

## Graphical abstract

Regional extinction rates can be higher or lower than local rates. Using simulations, we investigate the ecological mechanisms behind this apparent paradox. Specifically, we show that when the mortality of individuals decreases with population density, regional extinction rates are higher than local ones, and vice versa.

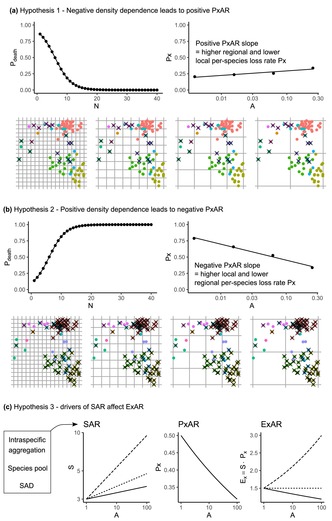

## 1 Introduction

Excessive loss of biodiversity via species extinctions and extirpations is a serious threat to human wellbeing and ecosystem functioning (IPBES 2019). Assessments of how fast and where species disappear are thus necessary to identify causes of the loss and for effective conservation decisions. The problem with such assessments is that both species diversity and its loss strongly depend on the area over which they are assessed.

When biodiversity is measured across multiple locations, for example, in a grid on a map, the average area of a location is *spatial grain* (hereafter *A*, Table 1). Average species diversity can only increase or remain constant with increasing grain (Arrhenius 1921; Storch 2016). However, there is a mounting empirical evidence that temporal change of diversity (McGill et al. 2015; Chase et al. 2019), rates of species gains (Sax and Gaines 2003), and extinction rates (Keil et al. 2018) can increase, decrease, or can have complex and non‐linear relationships with grain. Specifically, the average number of species that have disappeared from typical local patches (fine grain) may be higher or lower than the number of species that have disappeared from a typical large region (coarse grain). This has practical consequences: First, reports of biodiversity loss from a single grain can mask more or less dramatic losses at other grains. Second, analyses of the loss conducted at different grains are not comparable.

**TABLE 1:** Key terms and notation used in this paper. When we mention averages, we mean that the value was averaged across all grid cells (or locations) at a given grain *A*.

Symbol	Definition
*P*_death_	Probability that an individual of a species dies
*N*	Number of individuals of a single species in a region
*A*	Average area of a grid cell, which is also the spatial *grain*
*S*_1_	Average number of species in the area at time 1, i.e., before extinctions take place
*Ex*	Average number of species that are lost from grid cells of area *A*
*Px*	Average per‐species probability of extinction in area *A*, calculated as *Px* = *Ex/S*_1_
SAR	Relationship between *S*_1_ and *A*, also known as the nested species‐area relationship
SAD	Species‐abundance distribution
ExAR	Relationship between *Ex* and *A*
PxAR	Relationship between *Px* and *A*

Furthermore, the relationship between grain *A* and the rate of species loss depends on the metric of the loss. For example, the average number of species that went extinct (*Ex*, Table 1) can increase with increasing *A* (a relationship called ExAR; Table 1), while the average per‐species probability of extinction (*Px*, Table 1) can decrease with increasing *A* (a relationship called PxAR; Table 1), with all of this happening in the same region and taxonomic group (Keil et al. 2018).

Only a few studies explored the theoretical mechanisms behind this strong and complex grain dependency of diversity loss. Among the first were Cassey et al. (2006) who use a stochastic metacommunity model to link the magnitude of biodiversity change to different modes of species gains and to initial species spatial aggregation. Keil et al. (2018) also showed that the grain dependence of extinction rates should be widely expected, but they provided only a simplistic cartoon of the mechanisms behind it (in their Figure 2). This has recently been extended to also accommodate species gains (Leroy et al. 2024), but again, in a rather simple way. Yan et al. (2022) further explored how the relationship between local and regional extinction rates depends on the spatial distribution of species, and they found that it is affected by spatial aggregation and mean range size. However, so far, we lack theory linking the shape of the extinction rate–area relationship with specific ecological processes on the individual level.

In this paper we address this knowledge gap. We build on the idea in Figure 2 of Keil et al. (2018) which suggests that the direction (positive, or negative) of the relationship between per‐species extinction probability *Px* and grain *A* somehow depends on species' rarity. In this paper, we use the total number of individuals in a larger region (*N*) as the measure of rarity. The larger region is a relative term describing any region that is larger than the region in which local extinction rate is measured. Thus, the larger region can be an area only slightly larger than the local site, it can be a country, or a continent. Since we consider *N* to be proportional to range size (i.e., we assume that larger geographic ranges have higher *N*), the reasoning presented here can also apply to range size. Specifically, we propose that the per‐individual probability of death (*P*_death_) depends on the total number of individuals *N* (or range size), and this relationship can either be positive or negative, which will then lead to negative or positive relationship between grain and extinction rate *Px* respectively. This is what we call “density dependent rate of death” in the title, and we note that this density is calculated over the larger region, not locally. Here are specific hypotheses for how this density dependence can affect the extinction scaling:

1.1

**FIGURE 1:**
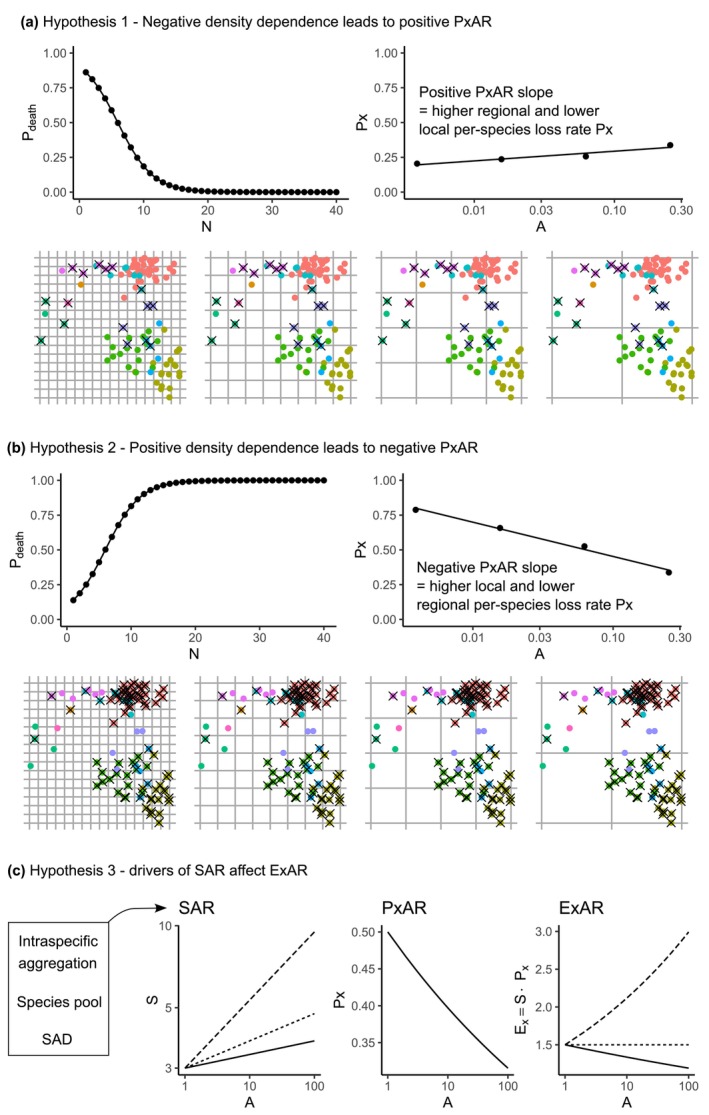
Three hypotheses of this study. Hypotheses 1 and 2 (a, b) predict different slopes of PxAR, which is the relationship between *per‐species* probability of extinction (*Px*) and area (*A*). (a) Negative density‐dependent mortality, that is, *per‐individual* probability of death (*P*_death_) decreases with the total number of individuals in a region (*N*), leading to a positive slope of PxAR. (b) Positively density‐dependent mortality, that is, *P*_death_ increases with *N*, leading to a negative slope of PxAR. Both hypotheses are illustrated on a point pattern in a square region; each individual is a dot, species are indicated by colors, and dead individuals are marked by a cross. The region is overlaid by four grids with increasing spatial grain *A*. (c) Hypothesis 3 states that the slope of ExAR, which is the relationship between the number of extinct species (*Ex*) and *A*, is affected by drivers of the slope of the species‐area relationship (SAR) before extinctions take place. These drivers are intraspecific aggregation, species pool, and the shape of the regional species‐abundance distribution (SAD).

Hypothesis 1 *When individuals of rare species (i.e., with low N or small range) are more likely to die than individuals of common species, there will be lower local and higher regional per‐species probability of extinction Px (Figure* 1a *)*.

This hypothesis assumes a *negative* relationship between the number of individuals (*N*) in a region, and the per‐individual probability of death (*P*_death_). As the number of individuals decreases, each of them is more likely to die (Figure 1a). One mechanism behind this can be Allee effect (Allee and Bowen 1932; Courchamp et al. 2009), which can occur, for example, in organisms that rely on intraspecific facilitation (Berec et al. 2007), in organisms that alter their environment to suit them better, or in populations susceptible to inbreeding depression or demographic stochasticity (Lande et al. 2003). Consequently, species with fewer individuals are more likely to go extinct than species with more individuals. A loss of a rare small population reduces the total number of species in a region, but not so much the average number of species in a local patch. Thus, under the (hugely) simplifying assumption that the negative density dependence applies to all species in a region, we expect local extinction rates to be lower than regional ones. In other words, the average *per‐species* probability of extinction (*Px*, Table 1) should increase with grain *A* (Figure 1a), that is, PxAR should have a positive slope.

Hypothesis 2 *If individuals of common species (i.e., with high N or large range) are more likely to die than individuals of rare species, there will be a higher local and lower regional per‐species probability of extinction Px (Figure* 1b *)*.

This hypothesis assumes a *positive* relationship between the number of individuals (*N*) in a region and the per‐individual probability of death (*P*_death_). The more individuals there are, the more likely each of them is to die. One mechanism behind this could be a simplified form of the Janzen–Connell effect (Connell 1970; Janzen 1970), which takes place when, for example, large population densities lead to increases in the densities of pathogens or other natural enemies, or in intraspecific competition, or when common species are an attractive catch for human hunters or fishers. Consequently, a decline in the population of common species (with high *N*) is unlikely to cause an extinction across the whole region (and once *N* declines to below a certain level, the effect no longer applies), but it should cause more frequent extinctions of the species from local patches, affecting local extinction rates more than regional ones. Thus, assuming the positive density dependence applies equally to all species in the community, the average per‐species probability of extinction (*Px*) should decrease with area *A* (Figure 1a), that is, PxAR should have a negative slope.

Hypothesis 3 *The relationship between the average counts of extinct species (Ex) and grain (we call this relationship ExAR; Table* 1 *) should also be affected by the density dependent mortality. However, ExAR also depends on the initial number of species (S), and hence it should be sensitive to variables affecting the slope of the initial species‐area relationship (SAR) in the region at time before extinctions take place, namely to species aggregation, the species pool, and the regional species‐abundance distribution (Figure* 1c *)*.

It is more difficult to predict how density‐dependent mortality affects ExAR. It holds that $E_{x}=S\times P_{x}$ at a given grain; here $P_{x}$ is the average, but the logic holds even if each species has a different probability of extinction (see the online repository for a demonstration). When $E_{x}=S\times P_{x}$, then *Ex* is affected both by *Px* and by the number of species (*S*) present before the extinctions take place. Species richness *S* follows a nested SAR (Storch 2016), and thus ExAR=SAR×PxAR. Therefore, ExAR should be affected both by the negative and positive density dependent mortality (through PxAR, if Hypotheses 1 and 2 hold), and by the slope of the *SAR*. The most important drivers of the SAR slope are *intra‐specific spatial aggregation*, size of the *species pool*, and the shape of the regional *species‐abundance distribution* (SAD) (Storch et al. 2008; McGlinn et al. 2019). We thus expect all these to affect the slope of the ExAR, in addition to the density dependence described in Hypotheses 1 and 2. However, the interplay can be complex, as even a simple monotonic PxAR and SAR can lead to a plethora of functional forms of ExAR, including nonlinear or hump‐shaped ExARs, as demonstrated by Keil et al. (2018).

## 2 Methods

We performed two simulation experiments (code and simulated data are available on Zenodo: https://doi.org/10.5281/zenodo.14720166). We tested all three hypotheses in each experiment. The first experiment is based on spatially explicit point patterns; it has two time steps (before and after the extinction) and hence it represents a non‐equilibrium community dynamics. The second experiment is based on a spatially implicit system of generalized Lotka–Volterra equations with many time steps and thus represents a community at equilibrium. As these two experiments are conceptually different, a result that emerges consistently in both can be seen as general, while differences among them can point to interesting ecological mechanisms. The reason we have chosen point patterns and LV simulations was based on our specific technical skills and prior experience with these kinds of models.

### 2.1 Point Pattern Experiment

**FIGURE 2:**
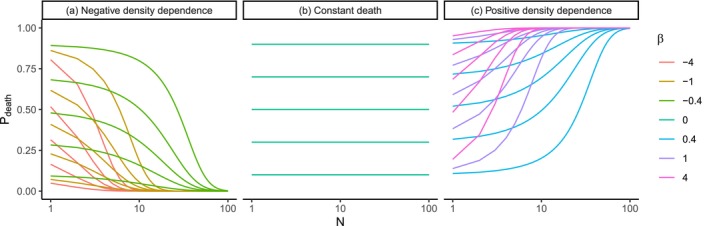
Illustration of how parameter β affects the shape of the Barták function (Equation 1). β<0 is the negatively density‐dependent *P*_death_ (left panel), β=0 is a density‐independent *P*_death_ with constant values for all population sizes (middle panel), and β>0 is a positive density‐dependence (right panel). The curves also vary in parameter α, which is the intercept of the Barták function. Note the log_10_ *x*‐axis.

We first tested our hypotheses on a set of simulated point patterns; these have the advantage of being spatially explicit and allowed testing Hypothesis 3 under varying total numbers of species, individuals, shapes of the initial species‐abundance distributions (SAD), and spatial aggregation of individuals, all of which affect the SAR (McGlinn et al. 2019). Spatial aggregation has also been shown to affect the spatial scaling of extinction rates (Yan et al. 2022). We performed the simulations in R (R Development Core Team 2022) using a combination of the ‘mobsim’ package (May et al. 2018) and our custom functions (complete code is in the Zenodo repository, https://doi.org/10.5281/zenodo.14720166). Specific parameters of the simulations are in Table 2. Each simulation had only 3 steps, which represent an initial state, death of individuals, and calculation of species loss (extinction rates):

1. *Species distributions in time 1.* The simulations took place in a square region with an area of 1 imaginary unit (as shown in Figure 1). We populated the region with a total number of *N*_*tot*_ points and *S*_*tot*_ species, using the Thomas point process, each species with a single mother point and displacement from the point given by the parameter *σ*. The number of individuals (*N*) of each species was given by a lognormal SAD whose evenness was set by a single parameter *CV*_*N*_ (explained in Table 2).
2. *Death of individuals.* In the next step, we subjected every individual in the region to death with probability *P*_death_, which was made density‐dependent according to a function:
(1) $$P_{\text{death}}\left(N,\alpha ,\beta \right)\;\begin{array}{ll} = & \lim_{b\to \beta }\frac{e^{N\times \mathrm{sgn}\left(b\right)\times e^{\left(-\frac{1}{\left|b\right|}\right)}}}{\frac{1-\alpha }{\alpha }+e^{N\times \mathrm{sgn}\left(b\right)\times e^{\left(-\frac{1}{\left|b\right|}\right)}}}\\ = & \left\{\begin{array}{ll} \frac{e^{N\times \mathrm{sgn}\left(\beta \right)\times e^{\left(-1/|\beta \right)}}}{\frac{1-\alpha }{\alpha }+e^{N\times \mathrm{sgn}\left(\beta \right)\times e^{\left(-1/|\beta \right)}}} & \text{for}\;\beta \neq 0\\ \alpha & \text{for}\;\beta =0 \end{array}\right. \end{array}$$
  where, $N\in \left\{1,2,\ldots \right\}$ is the total number of individuals of a species in the region. We call Equation (1) the Barták function. Equation (1) is a two‐parameter function: (1) Parameter $\alpha \in \left(0,1\right)$ is the “intercept”, that is, the probability of an individual's death when there is just a single individual in the region. (2) Parameter β∈ℝ is the “slope” of the Barták function, where β<0 makes the function decreasing (i.e., negative density dependent mortality), β>0 makes it increasing (i.e., positive density dependent mortality), and β=0 means that there is no relationship between *N* and *P*_death_ (Figure 2). Note that the Barták function is purely phenomenological, with no a priori biological or mechanistical meaning; we designed it so that the strength (and sign) of the density dependence of the death rate can be conveniently manipulated by β, but the same effect can likely be achieved using some other flexible function. To execute the actual deaths of the individuals, we subjected each individual point to a Bernoulli trial with *P*_death_ as its *P* parameter.

3 *Calculation of species loss.* Finally, we overlaid the region with grids of increasing resolutions, where we divided the region into 2 × 2, 4 × 4, 8 × 8, and 16 × 16 grid cells. At each resolution, we calculated *Ex* and *Px* (Table 1). We then fitted the PxAR as a linear regression of *Px* as a function of log(*A*), and ExAR as a Poisson generalized linear model (log link function) of *Ex* as a function of log(*A*).

**TABLE 2:** Parameters which we varied in the point pattern simulations.

Parameter	Description	Parameter values in the simulation
β	“Slope” of the Bartak function. β<0 means that *P*_death_ increases with *N*, i.e., positively density‐dependent mortality. β=0 means that *P*_death_ is independent on *N*. β>0 means that *P*_death_ decreases with *N*, i.e., negatively density‐dependent mortality.	−4, −1, −0.4, 0, 0.4, 1, 4
α	“Intercept” of the Bartak function. It is the probability of death when there is just a single individual of a species in a region. In the main text we present simulations which kept α constant across all species. In Appendix S1 we show simulations where α varied between species (with uniform distribution between 0.01 and 0.99).	0.01, 0.1, 0.3, 0.5, 0.7, 0.9, 0.99
*CV*_*N*_	Parameter that drives the evenness of the log‐normal species abundance distribution (SAD) in the region. It is the standard deviation of abundances divided by the mean abundance (no. of individuals/no. of species). *CV*_*N*_ is thus negatively correlated with the evenness of the SAD. Illustration of the effect of *CV*_*N*_ on SAD shape is in Figure 4c.	0.1, 1, 10
σ	Parameter that drives intra‐specific spatial aggregation of individuals. It is the mean displacement (along each coordinate axes) of a point from its mother point (= cluster center).	0.01, 0.1, 1
*S*_*frac*_	Average number of individuals per species. This is a parameter that determines the total number of species $S_{\mathrm{tot}}$ (a.k.a. species pool) in the simulation, since $S_{\text{frac}}=N_{\mathrm{tot}}/S_{\mathrm{tot}}$.	0.05, 0.1, 0.2
*N*_*tot*_	Total number of individuals in a region.	100, 1000

We repeated the three steps above for 2646 combinations of total number of individuals, size of species pool, shape of SAD, intra‐specific spatial aggregation, and slope and intercept of the Barták function (Table 2). Each combination was repeated 10 times, giving us 26,460 simulation runs. These simulations represent a scenario where each species follows the same shape of the Barták function; to test what would happen if we relaxed this, we did a set of simulations where species differed in the intercept α. Specifically, each species in a simulation was assigned α drawn from a uniform distribution between 0.01 and 0.99. We found that this did not qualitatively affect the results (Appendix S1, Figures S1 and S2), and so we only present the simulations with constant α here in the main text.

### 2.2 Lotka–Volterra Experiment

To complement the point pattern simulations, we tested our hypotheses using a spatially implicit metacommunity simulation where abundances of species were traced in a set of *M* local communities, each following a disordered systems model (described below) comprising a series of generalized Lotka–Volterra equations (Barbier et al. 2018). Notably, the shape of the species‐abundance distribution (SAD) was not specified à priori (as in experiment 1), but it emerged from the simulation. Thus, the magnitude of the density dependence of death rate (parameter *d*_*z*_, Table 3, similar to the β parameter for the point pattern simulations) affected the resulting SAD. We expected this to affect the resulting scaling: For example, in case of negatively density‐dependent mortality, rare species are constantly eliminated in each simulation step; over the long run, this should result in an even SADs with less rare species, and thus less extinctions, which could potentially “switch off” Hypotheses 1 and 2.

**TABLE 3:** The key parameters that we varied in the Lotka–Volterra simulations. There are additional parameters that we kept constant; they are listed in the documented code's repository on Zenodo: https://doi.org/10.5281/zenodo.14720166.

Parameter	Description	Parameter values in the simulation
*d*_*z*_	Parameter regulating the strength and sign of the density dependence of death rate. Specifically, it's a process noise nugget which determines how *P*_death_ relates to *N*. *d*_*z*_ < 2 means that *P*_death_ increases with *N*. *d*_*z*_ = 2 means that *P*_death_ is independent on *N*. *d*_*z*_ > 2 means that *P*_death_ decreases with *N*.	1, 1.2, 1.4, 1.6, 1.8, 2, 2.2, 2.4, 2.6, 2.8, 3
*M*	Number of local communities in a metacommunity.	10, 100
*μ*_*α*_	Mean strength of the interaction among species. For the sake of stability of the simulations, we always used negative mean interaction strength.	−0.9, −0.5, −0.1
*S*	Initial number of species (species pool) in the region.	10, 100

One Lotka–Volterra simulation began with an *initial state*, which was set as follows: There are *M* patches, with a total of *S* species. Each species *i* has a carrying capacity (*K*_*i*_), per‐capita growth rate (*r*_*i*_), and interspecific interaction strengths describing the per‐capita effect of each species *j* on species *i* (*α*_*j*,*i*_). These are drawn from standard normal distributions, with means *μ*_*K*_, *μ*_*r*_, and *μ*_*α*_, and standard deviations *σ*_*K*_, *σ*_*r*_, and *σ*_*α*_, respectively (with minimum value 0 for *K*_*i*_—note that *α*_*j*,*i*_ can be positive or negative). For simplicity, we assume that *α*_*j*,*i*_ and *α*_*i*,*j*_ are drawn independently. After the initialization, the simulation proceeded as a sequence of alternating dispersal and extinction events:

1. *Dispersal.* Species disperse across *M* patches, with dispersal and patch‐level disturbance events modeled as a series of discrete stochastic perturbations to the underlying Lotka–Volterra models, with exponentially distributed waiting times between events. Each species disperses to new patches with rate *πc*_*i*_, where *π* is the fraction of total patches occupied by species *i* (i.e., as in the classic Levins model; Levins 1969). After colonizing a new patch, the initial abundance of the arriving species, *n*_0,*i*_, is drawn from normal distributions with mean *μ*_*c*_ and *μ*_*n*0_, standard deviations *σ*_*c*_ and *σ*_*n*0_, and minimum value 0. We set *n*_*0*,*i*_ of all species to the absolute value of the random draw (to avoid negative abundance).
2. *Extinction.* Local extinction of a species within a local patch *m* (i.e., abundance of species *i*, $n_{\mathrm{im}}\left(t\right)=0$) occurs either because of species interactions, or from impacts of disturbances. Disturbances impact all species and patches simultaneously, with the average waiting time between disturbances *d*_*w*_. Disturbance events are drawn from a normal distribution with mean *μ*_*d*_ = 0 and standard deviation $\sigma_{d}=\sqrt{d_{c}n_{i,m}^{d_{z}}}$, where $n_{i,m}$ is the abundance of species *i* in patch *m*, and *d*_*c*_ and *d*_*z*_ are constants. Disturbances therefore impart stochastic structure to population dynamics that follow a Taylor Power Law (Taylor 1961). Thus, disturbance intensity grows as a nonlinear function of abundance, such that $d_{z}<2$ implies that disturbances are more likely to drive rare species to local patch‐level extinction (because $\sigma_{d}$ grows slower than $n_{\mathrm{im}}$, implying that relative impacts of disturbances decline with population size), whereas $d_{z}>2$ implies that common species are more likely to be driven to local extinction (because $\sigma_{d}$ grows faster than $n_{\mathrm{im}}$, implying that the relative impacts of disturbances increase with population size).

We tested all combinations of parameter values in Table 3, each repeated 10 times, giving us 1320 simulations in total. Each simulation was run for 20 steps (each involving dispersal and extinction). We chose 20 steps as it made the system of equations still possible to be solved in reasonable time; also, after examining several simulations, they tended to stabilize after roughly 10 steps.

In each simulation, we took a snapshot of species composition in all patches in step 10 and 20. A species was considered extinct in a patch (or in the whole metacommunity) if it was present in time 10 and absent in time 20. To calculate PxAR and ExAR, we calculated the average ${\mathrm{Px}}_{\text{patch}}$ and ${\mathrm{Ex}}_{\text{patch}}$ across all patches, and ${\mathrm{Px}}_{\text{metacom}}$ and ${\mathrm{Ex}}_{\text{metacom}}$ at the metacommunity scale. The slope of the PxAR was then $\left({\mathrm{Px}}_{\text{metacom}}-{\mathrm{Px}}_{\text{patch}}\right)/t$ and the slope of ExAR was $\left({\mathrm{Ex}}_{\text{metacom}}-Ex_{\text{patch}}\right)/t$, where t=10, which is the time between the two measurements.

### 2.3 Evaluating the Simulations

For each simulation we noted the parameter values (Tables 2 and 3) as well as the slope of the PxAR and ExAR. We then plotted the slope of the PxAR and ExAR as a function of the parameter β (in point patterns simulations), or as a function of parameter *d*_*z*_ (in Lotka–Volterra simulations).

To compare the effects of different simulation parameters on the slope of PxAR and ExAR, we conducted a variable importance analysis using the Random Forest algorithm (Hastie et al. 2011) with the R package randomForest (Liaw and Wiener 2002) in which the slope of the PxAR or ExAR was the response variable, and parameters of the simulation (Tables 2 and 3) were the predictors. For the hyperparameters, we used the default settings of the randomForest R function (500 trees, 1/3 of predictors and 63% of observations sampled in each bag, terminal node size of 5). We note that it makes little sense to evaluate the importance of simulation parameters using *p*‐values (White et al. 2014). We thus make our inference by directly comparing the frequency distributions of PxAR and ExAR slopes or the variable importance from the random forest analyses.

## 3 Results

**FIGURE 3:**
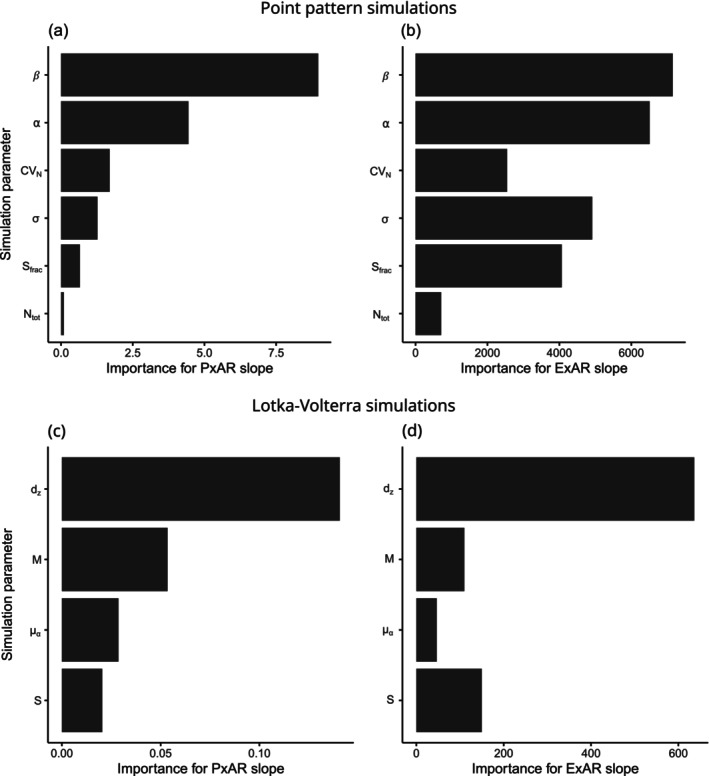
Importance of (a, b) point pattern, and (c, d) Lotka–Volterra simulation parameters for determining the slope of (a, c) PxAR and (b, d) ExAR. For explanation of parameters, see Tables 2 and 3. The importance is measured as the total decrease in node impurities from splitting on a given predictor variable, averaged over all trees in a random forest analysis.

In both experiments, and among all other parameters considered, the parameters that controlled the magnitude of density‐dependent mortality (*β* or *d*_*z*_) had the most important effect on slopes of both PxAR and ExAR (Figure 3). In the point pattern simulations, the single‐individual *P*_*death*_ (set by the parameter *α*) was also important. This was the case when α and β were the same for all species in a simulation run (Figure 3), but also when we varied α among species (Appendix S1, Figure S1).

In the Lotka–Volterra experiment, the slope of the ExAR was also sensitive to the species aggregation (parameter *σ*) and the average species abundance (parameter *S*_fraq_).

### 3.1 Hypothesis 1

**FIGURE 4:**
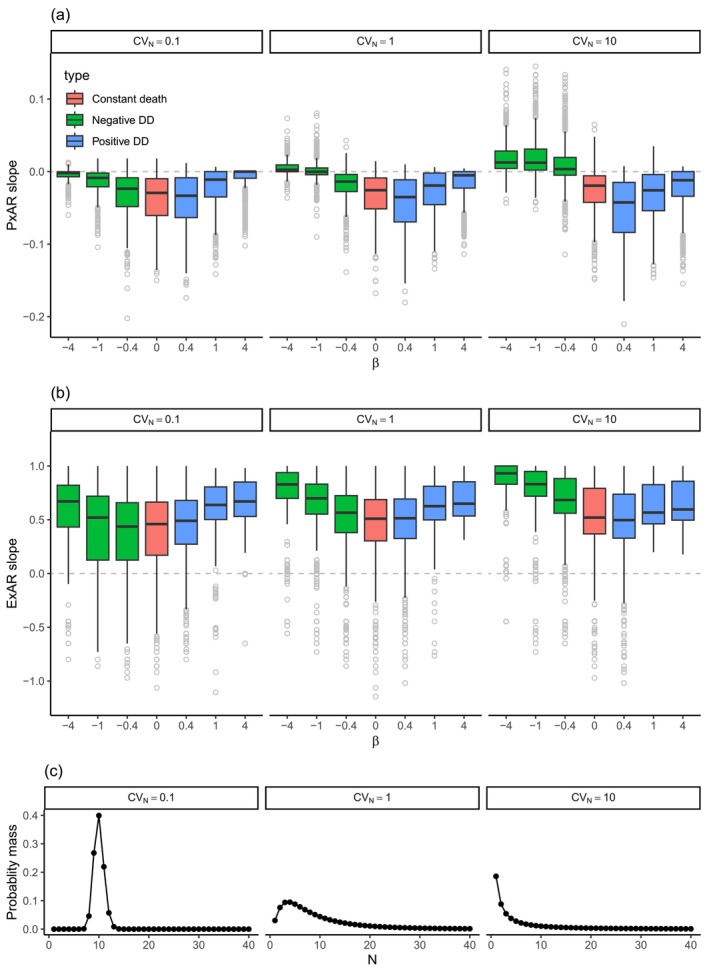
The effect of the sign and magnitude of density‐dependence of death rate (determined by parameter β, Table 2) on the slope of PxAR (a) and ExAR (b) in point pattern simulations. Panels are divided according to three levels of the *CV*_*N*_ parameter, which affects the shapes of the regional species‐abundance distribution (SAD), from more even (left) to uneven (right). These SADs are illustrated in panel (c).

We found that presence of the negative density dependence of mortality in the point pattern simulations led to positive slopes of PxAR (Figure 4a, green boxes), consistent with Hypothesis 1, but only when there were species with relatively low *N* in the regional SAD (i.e., *CV*_*N*_ was high and the SAD was uneven) and when α was high enough to cause enough mortality (Appendix S1, Figure S3). For even SADs (*CV*_*N*_ = *0.1*), the negative density dependence produced a PxAR slope that was higher than a constant *P*_death_ scenario (Figure 4a, red box), but often negative. Additionally, for these even SADs, the PxAR slope approached zero as the density dependence became more severe (i.e., extreme negative values of β), which reflects generally low mortality in these scenarios.

**FIGURE 5:**
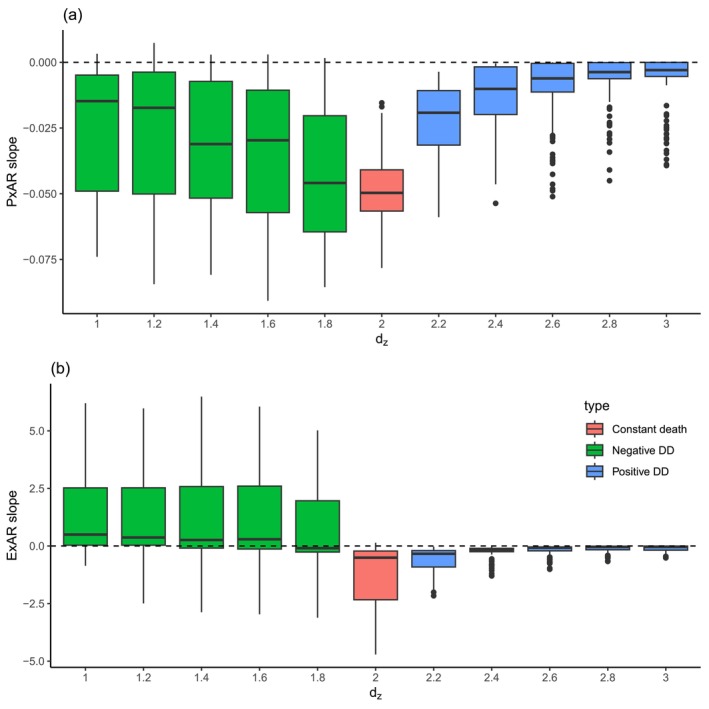
The effect of parameter *d*_*z*_ on the slope of PxAR (a) and ExAR (b) in Lotka–Volterra simulations. The parameter *d*_*z*_ regulates the sign and magnitude of the density dependence of the death rate.

In the Lotka–Volterra simulations, we did not find support for Hypothesis 1, although simulations with negative density dependence were the only ones that produced the rare positive PxAR slopes (Figure 5a).

### 3.2 Hypothesis 2

We found that positive density dependence of mortality in point pattern simulations produced negative slopes of PxAR (Figure 4a, blue boxes), consistent with Hypothesis 2. This was more pronounced when evenness of the regional SAD (parameter *CV*_*N*_) was low. In such cases, the slope of the PxAR was more negative than a slope expected from constant *P*_death_ (red box in Figure 4a when *CV*_*N*_ = *10*). In all other cases in the point pattern, the PxAR slope under the positive density dependence was similar to the slope of the constant *P*_death_, with some variation depending on parameter α. Specifically, high values of α and β caused extremely high mortality, pushing the PxAR slope to 0, as most individuals died at all scales.

In the Lotka–Volterra experiment, however, the PxAR slope was negative (as expected under Hypothesis 2), but higher than in the constant *P*_death_ scenario (Figure 5a). As in the point pattern simulation, the stronger was the positive density dependence (the more parameters β and $d_{z}$ increased), the more the PxAR slope approached 0 (Figure 5a), again likely reflecting extremely high mortality rates in these scenarios.

### 3.3 Hypothesis 3

As expected, ExAR was affected by different parameters than PxAR (Figure 3). In line with Hypothesis 3, the total number of species (*S*_*frac*_ and *S*) had a stronger effect on ExAR than on PxAR in both point pattern and Lotka–Volterra simulations. In addition, in the point pattern simulations (Figure 3a,b), the ExAR (but not PxAR) slope was strongly affected by the evenness of the regional SAD (parameter *CV*_*N*_) and by con‐specific spatial aggregation (parameter *σ*), also in line with Hypothesis 3.

## 4 Discussion

To get back to our title question: Should regional species loss be faster or slower than local loss? Our main finding is that this depends on the strength of the relationship between the number of individuals in the region (rarity) and the per‐individual probability of death (mortality). This role of rarity is even more important than other drivers of the extinction scaling, such as conspecific aggregation. Particularly, the latter was pointed out as an important driver of extinction scaling by (Yan et al. 2022). We confirm Yan et al.'s (2022) results, but we also extend them by adding an even more important mechanistic driver than a simple conspecific aggregation.

Another important finding is the difference between the spatially explicit point pattern simulations and the Lotka–Volterra simulations. There was a general scarcity of the positive PxAR (lower local and higher regional per‐species extinction rates), which we only invoked in the point pattern simulations, particularly in uneven initial SAD. In other words, although Hypothesis 2 was generally confirmed, Hypothesis 1 was confirmed only in the point pattern simulations. Here is our explanation: With the point patterns, we simulated a *one‐time loss event*, in which rare or common species were present at time 1, and the density‐dependent mortality could target these rare or common species, leading to extinction scaling that was in line with our hypotheses. However, the Lotka–Volterra simulations represented a system at a steady state where rare or common species were constantly targeted in every step of the simulation, thus leading to an elimination of rarity or commonness from the system, making the SAD more even and the PxAR negative.

Examples of the one‐time loss events are abrupt human‐caused changes in landscape management, land cover, disturbances such as fires, or extreme climate events. We expect these to be generally density‐independent causes of mortality affecting all individuals equally (corresponding to the constant death scenario), causing higher local and lower regional (or global) per‐species probability of extinction. On the other hand, real‐world examples corresponding to a large‐scale negative density dependence (Hypothesis 1) are global extinctions of endemic species from islands (Loehle and Eschenbach 2012) caused by the arrival of humans (Nogué et al. 2021) who introduced invasive species such as rats and domestic cats. Small island populations of endemic species were no match for the introduced predators, making them exceptionally vulnerable to extinctions, but these extinctions had little effect on local species counts in the rest of the world. This caused a positive global PxAR, with high global and low local per‐species probability of extinction. An example of human pressure that causes positive density‐dependent mortality (Hypothesis 2) is whaling and fishing, which disproportionally affect abundant species and schools of fish. Most of the species still survive somewhere on Earth (Barnosky et al. 2011), but numerous stocks have collapsed (Pinsky and Byler 2015), likely leading to higher local but lower global per‐species extinction rates.

In stable systems closer to a steady state where mortality is a continuous process, we expect a generally negative PxAR, irrespective of whether the density dependence is negative or positive. This is also what has been observed in such systems, for example, in a long‐term time series of Czech birds, or in the Barro–Colorado forest plot (Keil et al. 2018).

Concerning the total number of extinct species, and in line with Hypothesis 3, the direction of the ExAR was affected by other important drivers, that is, other than by the relationship between per‐individual death rate and rarity, specifically by factors affecting the SAR. As a consequence, the observed slopes of ExAR were variable and not correlated with PxAR slopes. This is also what has been broadly observed in empirical data where both negative and positive ExARs are common (Keil et al. 2018).

Some may argue that our exercise only involved limited numbers of individuals, which may correspond to a small spatial extent. This may be realistic in the context of local plots (e.g., forest plot dynamics; forestgeo.si.edu), but over regional and continental extents, total abundances of real‐world species are likely orders of magnitude higher. Further, we tentatively suggest that our results are still relevant for large extent situations involving species' geographic ranges; one simply can replace point occurrence with occupied grid cells across a continent, and one can replace per‐individual probability of death with per‐grid cell probability of loss of the species per grid cell. Similar principles of extinction scaling should then apply both for grid cell occupancy and our point pattern simulations. Moreover, once we consider occupancy, the same principles also apply for organisms for which individuals are not well defined, such as some plants. This connection between presence and absence of individuals, abundance, and species occupancy across scales has been described in the form of the occupancy‐area relationship (Kunin 1998).

In our main simulations, we assumed that the negative or positive density‐dependent mortality applies to all species in the community, while real‐world species likely differ in susceptibility to these effects, or both types can operate within a single system. However, when we relaxed this assumption partially by varying the baseline mortality by species (Appendix S1, Figures S1 and S2), we found that the results were similar to the scenarios with a constant form of density dependence. This is encouraging, as our results can perhaps be generalized outside of our controlled virtual setting.

We envision several follow‐ups: First, a similar exercise can be done for the rates of species gain. Recently, Leroy et al. (2024) proposed a simple idea that distinguishes local gain caused by within‐regional homogenization from gain caused by colonization from an outside pool, and how this affects the spatial scaling of species gain. A quantitative evaluation of such a proposition, in the spirit of the simulations presented here, would be a logical next step. Second, a mechanism could be tested that also includes spatial inter‐specific dependence of the probability of death among individuals. This can either be caused by spatially auto‐correlated mortality events such as fires, floods, or pest outbreaks, or by the loss of species that maintain vital mutually beneficial interactions. A loss of one species can thus lead to a domino effect in which other species are lost, maybe with interesting consequences for the scaling of the loss. Finally, the simulations can be made more realistic. For instance, many more simulation parameters can be varied, and their effect on the results examined, although without a clear prior expectation of their effect, this could be a never‐ending struggle. Another step towards realism can be to do a similar exercise over spatial extents and grains that more closely resemble continents or the entire world; this would follow developments of other scaling theories that were initially developed using extremely simplified local simulations (e.g., Hubbell 2001) and later extended to be geographically realistic (Rosindell and Cornell 2007).

In all, we see the main value of this exercise in the strengthening of the argument that biodiversity loss and biodiversity change in general must be considered as a strongly scale‐dependent problem. This has so far been acknowledged based on numerous empirical observations (Keil et al. 2011, 2018; Powell et al. 2013; Chase et al. 2019; Leroy et al. 2023). Here, by linking individual demographic parameters to the community level, we add a direct ecological mechanistic interpretation of this scale dependence.

## Author Contributions

**Petr Keil:** conceptualization (equal), data curation (equal), formal analysis (equal), funding acquisition (equal), investigation (equal), methodology (equal), project administration (equal), resources (equal), software (equal), supervision (equal), validation (equal), visualization (equal), writing – original draft (equal), writing – review and editing (equal). **Adam T. Clark:** methodology (equal), software (equal), writing – review and editing (equal). **Vojtěch Barták:** methodology (equal), software (equal), writing – review and editing (equal). **François Leroy:** conceptualization (equal), investigation (equal), methodology (equal), software (equal), visualization (equal), writing – review and editing (equal).

## Conflicts of Interest

The authors declare no conflicts of interest.

## Supporting information


Appendix S1.

## Data Availability

All code, simulated data, and figures are available in a Zenodo repository at https://doi.org/10.5281/zenodo.14720166.
