# Supplementary material for: Should Regional Species Loss Be Faster or Slower Than Local Loss? It Depends on Density‐Dependent Rate of Death
Source: Ecol Evol. 2025 Mar 27;15(4):e71162. doi: 10.1002/ece3.71162 (PMC11949570; doi:10.1002/ece3.71162)
Supplement: Supplementary file 1 — Appendix S1. [file ECE3-15-e71162-s001.pdf]

Appendix S1

Title: Should regional species loss be faster, or slower, than local loss? It depends on density-dependent rate of death

Journal: *Ecology and Evolution*

Authors: *Petr Keil, Adam T. Clark, Vojtěch Barták, François Leroy*

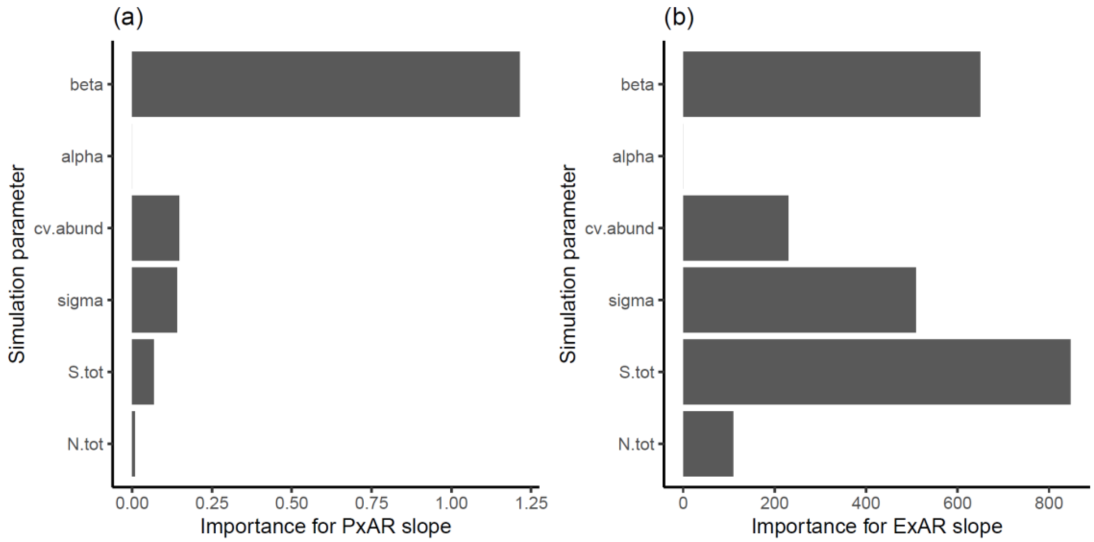

Figure S1 Importance of the point pattern simulation parameters for determining the slope of (a) PxAR and (b) ExAR in extra simulations where *we varied parameter α by species* (for each species it was drawn from a uniform distribution between 0.01 and 0.99). For explanation of parameters see Tables 2 and 3. The importance is measured as the total decrease in node impurities from splitting on a given predictor variable, averaged over all trees in a random forest analysis.

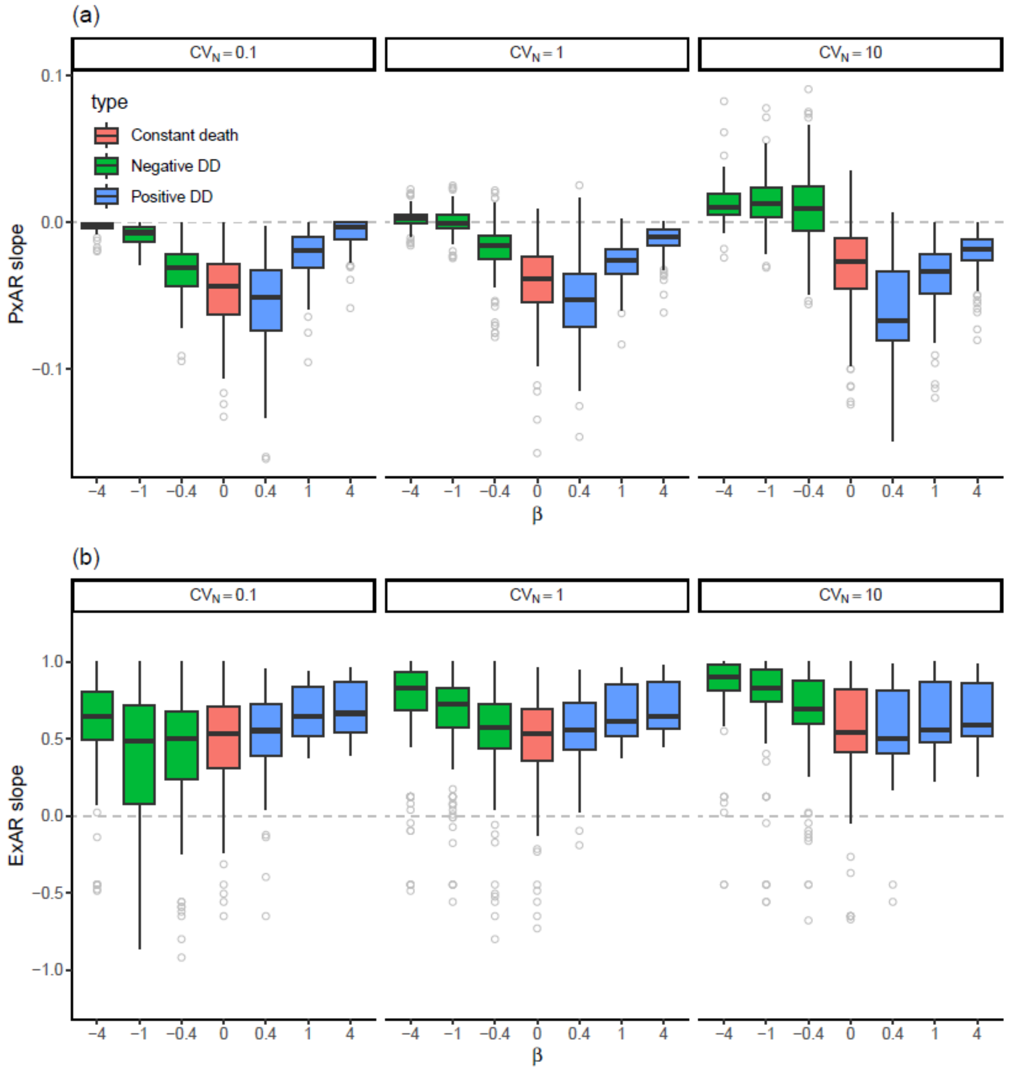

Figure S2 The effect of the sign and magnitude of density-dependence of death rate (determined by parameter β, Table 2) on the slope of PxAR (a) and ExAR (b) in extra point pattern simulations in which *we varied parameter α by species* (for each species it was drawn from a uniform distribution between 0.01 and 0.99). Panels are divided according to three levels of the CV_N parameter, which affects the shapes of the regional species-abundance distribution (SAD), from more more even (left) to uneven (right); see Fig. 4c of the main text.

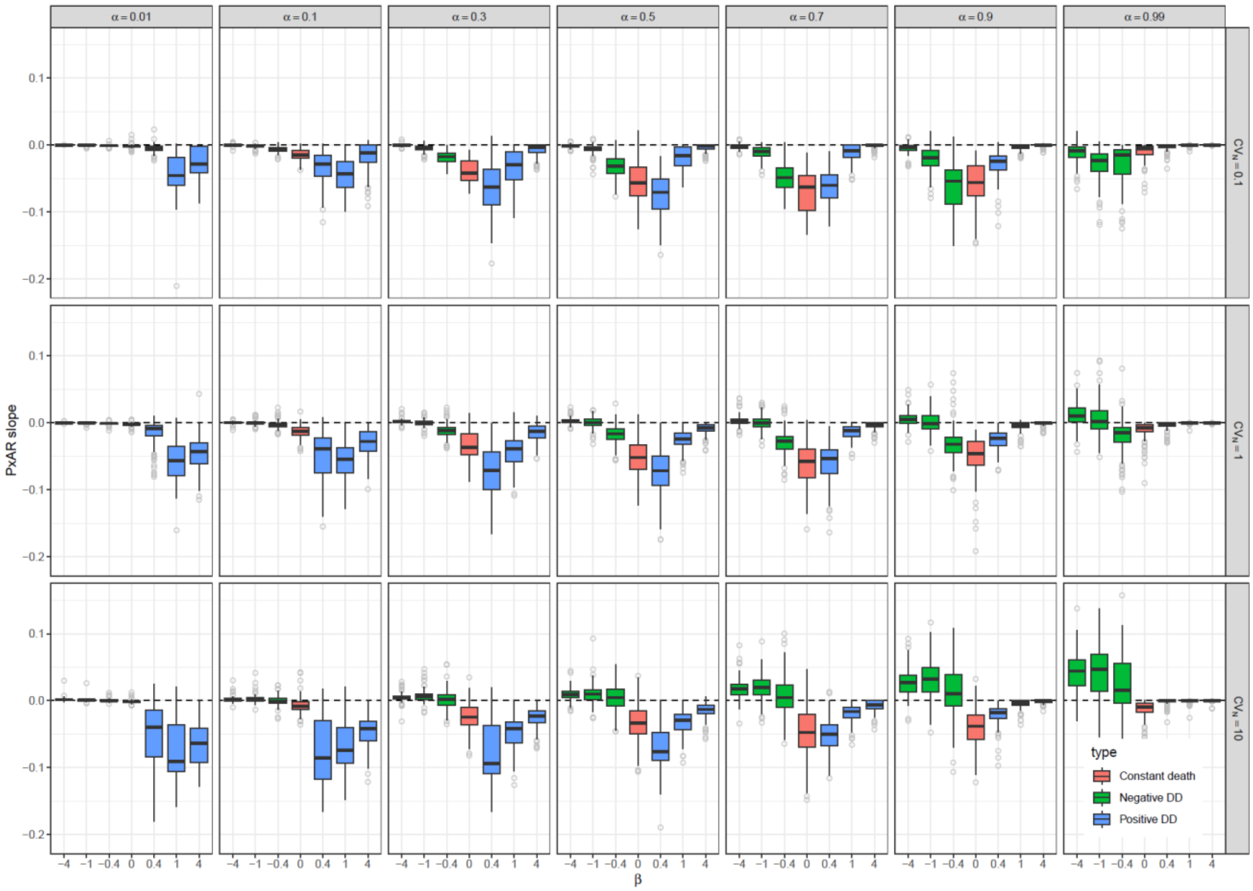

Figure S3 The effect of the sign and magnitude of density-dependence of death rate (determined by parameter β) on the slope of PxAR in the main point pattern simulations, and its interaction with parameters CV_N (shape of the SAD) and α (the intercept of the Barták function).

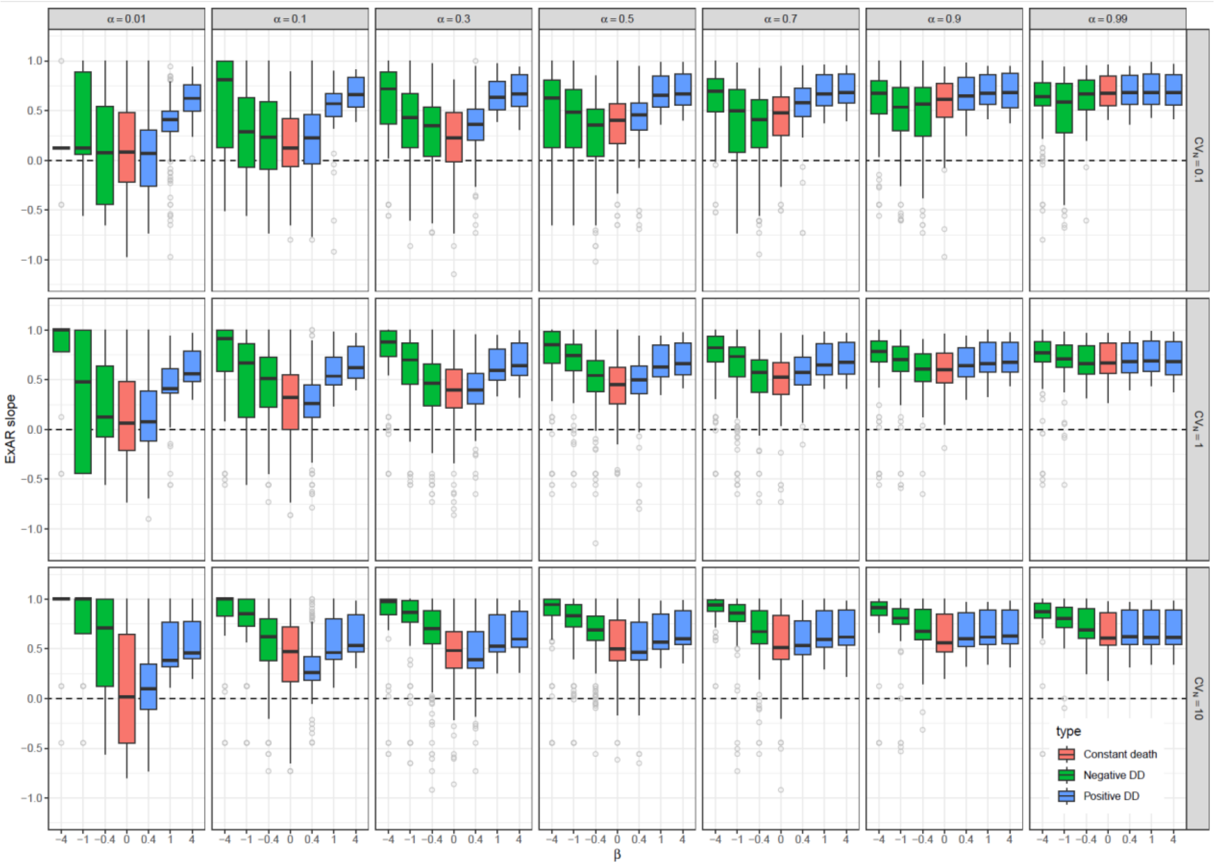

Figure S4 The effect of the sign and magnitude of density-dependence of death rate (determined by parameter β, Table 2) on the slope of ExAR in the main point pattern simulations, and its interaction with parameters CV_N (shape of the SAD) and α (the intercept of the Barták function).
